# Synthetic utility of oxygenases in site-selective terpenoid functionalization

**DOI:** 10.1093/jimb/kuab002

**Published:** 2021-01-25

**Authors:** Hans Renata

**Affiliations:** Department of Chemistry, The Scripps Research Institute, 130 Scripps Way, Jupiter, FL 33458, USA

**Keywords:** Terpenoid, C–H hydroxylation, Oxidation, P450

## Abstract

Terpenoids are one of the largest classes of natural products whose members possess a wide variety of biological activities. With several exceptions, scalable production of complex terpenoids with either purely biological or chemical methods still remains a major challenge. However, recent efforts to combine the two approaches in chemoenzymatic synthesis hold tremendous promise to address this challenge. Central to this paradigm is the development of useful biocatalytic methods, such as regioselective C–H oxidation, for terpene modifications. This review highlights recent applications of biocatalytic hydroxylation for site-selective modification of terpenoids.

## Introduction

**Fig. 1.**
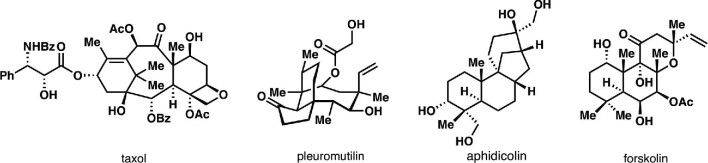
Selected examples of bioactive terpenes that have found use as medicine or biochemical tools.

Comprising more than 50 000 known members, terpenoids constitute one of the largest classes of natural products (Breitmaier, 2006). Members of this class arise biosynthetically from controlled cyclization of linear polyisoprene precursors catalyzed by various terpene cyclases, followed by peripheral modifications of the corresponding cyclized products through the action of various tailoring enzymes. Enriching the structural and chemical diversity of this class further, certain terpenoids—termed as meroterpenoids—are produced in nature from a mixed biosynthetic pathway involving a combination of isoprene-based and amino acid/polyketide/alkaloid-based building blocks. In addition to their structural diversity, terpenoids have long been recognized for their utility as medicinal agents. The highly oxidized diterpenoid taxol (Weaver, 2014) is a potent microtubule stabilizer that is currently in use clinically as chemotherapy medication (Fig. 1). Retapamulin (Jones et al., 2006), an antibacterial agent used for the treatment of impetigo, emerges from medicinal chemistry efforts to optimize the properties of the diterpenoid pleuromutilin. Other terpenoids such as aphidicolin (Ikegami et al., 1978) and forskolin (Alabashi & Melzig, 2012) have also found use as biochemical tools in physiological studies.

The high fraction of sp^3^-hybridized atoms and three-dimensionality of many cyclic terpenoids allow them to bind to their protein targets in a highly specific manner. However, these properties also constitute a double-edged sword as they pose significant hindrance for de novo preparation of these compounds. Large-scale production of bioactive terpenoids to meet supply demand has mostly relied on semisynthetic derivatization of natural materials or fermentation-based strategies. For example, taxol is currently produced via plant cell fermentation technology (Tabata, 2004) employing cell cultures of *Taxus chinensis* and topical corticosteroid drugs are obtained from chemical modifications of naturally isolated steroid hormones. While advances in synthetic biology technology have permitted the production of synthetic artemisinin with comparable cost to biomass extraction (Paddon et al., 2013), such success stories remain far and few between. Some of the major drawbacks (Pfleger & Prather, 2015) in this area are the relative lack of gene clustering in plant genomes and the need to optimize a myriad of parameters, such as the catalytic efficiency of enzymes involved and metabolic fluxes, to attain useful titers of compound production. Moreover, pathway engineering for the production of unnatural derivatives is still a challenging endeavor.

Despite challenges associated with step and atom economy, chemical synthesis for natural products offer the potential to generate unnatural derivatives once a modular route is established. Methodology developments have also advanced rapidly in recent years to allow many complex molecules to be constructed in an efficient manner. One modality in this area is the development of methods for direct functionalizations of C–H bonds in organic molecules (Yamaguchi et al., 2012). This strategy holds enormous potential as it offers the possibility of bypassing unnecessary functional group interconversions and other concession steps in chemical synthesis. Given the myriad of oxygenases (Barry & Challis, 2013; Hausinger, 2004; Podust & Sherman, 2012; Rudolf et al., 2017) that have evolved naturally, biocatalytic methods provide complementary approach for such functionalization of terpenoids and in some cases, also prove advantageous in addressing unmet regioselectivity, stereoselectivity, and chemoselectivity challenges. This review outlines recent developments in biocatalytic methods for C–H hydroxylation of terpenoids. The chosen case studies have been divided according to whether the enzymatic hydroxylation approach was performed for late-stage modification or for building block synthesis. In the former, an advanced intermediate, initially obtained either through isolation from natural sources or chemical synthesis, is subjected to regiodivergent oxidative functionalization with a suite of oxygenase enzymes. Conversely, the latter approach involves the production of a key early intermediate through enzymatic oxidation, which is then subjected to further transformations to produce the desired terpenoid products. Critical discussion is presented in each section to assess the impact of the work within the context of terpenoid synthesis and modification. Finally, some concluding thoughts on future directives of this approach for terpenoid synthesis are provided at the end of the review.

## Late-Stage Derivatization

### Divergent Oxidation of Artemisinin

**Fig. 2.**
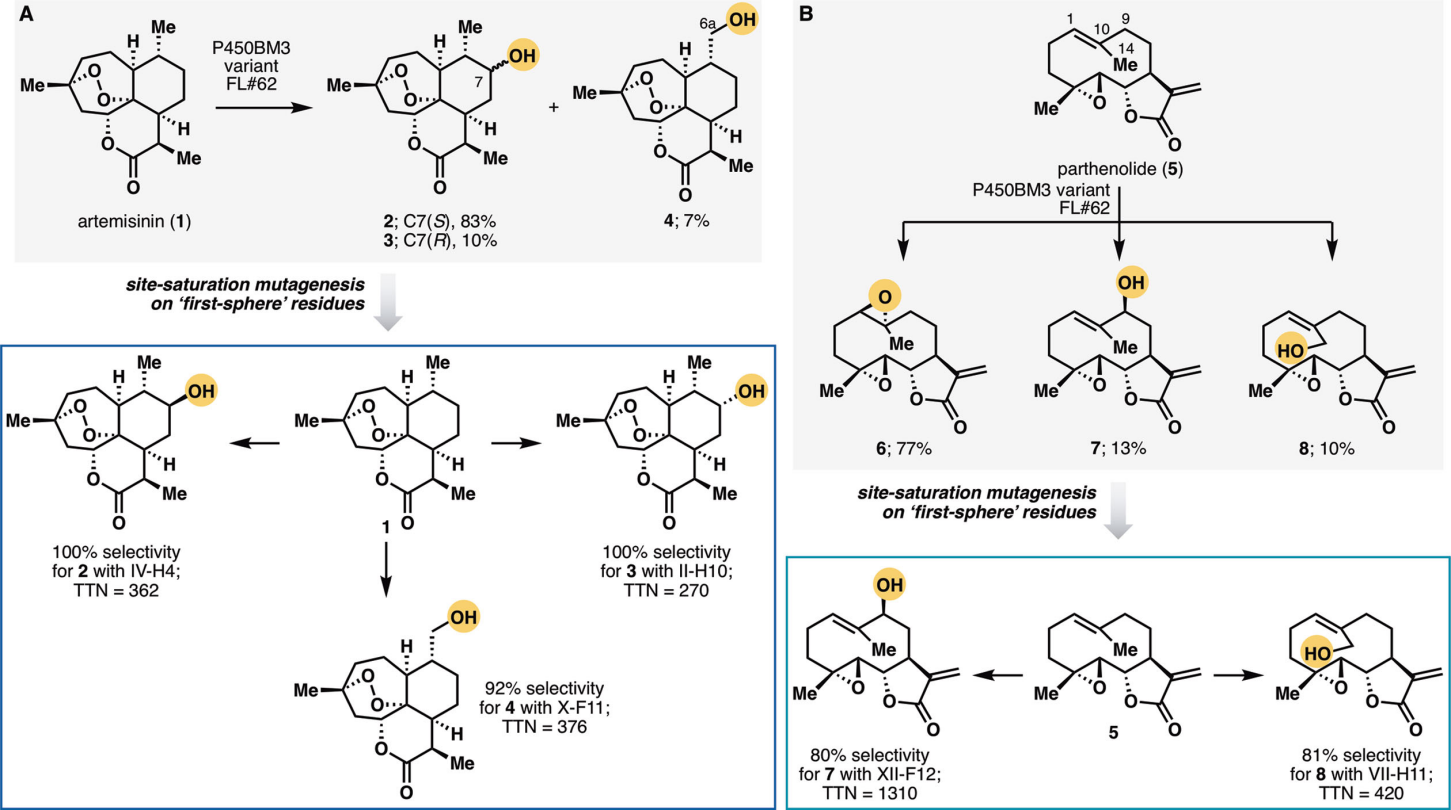
(A) Engineering of P450BM3 variant FL#62 for the discovery of biocatalysts for site-selective oxidation of artemisinin (**1**). (B)**.** Engineering of P450BM3 variant FL#62 for the discovery of biocatalysts for site-selective oxidation of parthenolide (**5**).

The sesquiterpene lactone artemisinin has been used widely as a standard treatment for *Plasmodium falciparum* malaria (White, 1997). Though initially obtained through direct isolation from the plant *Artemisia annua*, recent advances in synthetic biology and chemical synthesis have identified feasible strategies for synthetic production of artemisinin (Paddon et al., 2013). Despite its efficacy against malaria, artemisinin exhibits poor solubility in oil and water, thereby limiting its intravenous use in the treatment of advanced malaria cases. Efforts to address this limitation have led to the development of structural derivatives including the compounds artemether, arteether, and sodium artesunate. As a complementary strategy to obtain improved antimalarial agents, the Fasan laboratory developed an enzyme engineering strategy to identify enzymes that are capable of hydroxylating artemisnin (**1**) in a site-selective manner (Zhang et al., 2012). Prior work from the Fasan laboratory has shown the feasibility of a fingerprinting-based directed evolution strategy to optimize the hydroxylation activity of P450BM3 (CYP102A1) mutants on simple terpenoids (Zhang et al., 2011). Additionally, this work also led to the discovery of an engineered variant called FL#62, which shows efficient hydroxylation activity against a broad range of substrates. FL#62, in particular, was found to produce a mixture of three products, **2, 3**, and **4** in 83 : 10 : 7 ratio upon reaction with artemisinin (Fig. 2A). Starting from FL#62, the authors then performed site-saturation mutagenesis of “first-sphere” active site residues in search of selective P450 catalysts for the hydroxylation of artemisinin. In combination with multivariate analysis, this initial campaign resulted in several distinct P450BM3-based catalysts, which show remarkable site-selectivity for the production of C7(*S*)-OH (**2**) and C7(*R*)-OH (**3**) products, respectively. Notably, variants providing divergent stereochemical outcome for C7 hydroxylation could readily be obtained using this engineering strategy. Another variant, II-E2, predominantly forms the C6a-OH product (**4**), though with suboptimal regioselectivity. Further triple site-saturation mutagenesis on II-E2 improved the regioselectivity toward C6 hydroxylation significantly, resulting in a variant called X-F11 that is capable of forming **4** with 92% regioselectivity. The authors further showed that these biocatalytic transformations could be performed on preparative scale and that the resulting products could be employed in further derivatization to produce 7(*R*)-fluoroartemether and 7(*R*)-fluoroartesunate.

### Divergent Oxidation of Parthenolide

Parthenolide (**5**) is a naturally occurring sesquiterpene lactone that has been shown to affect several signaling pathways through covalent engagement by its α-methylene-γ-butyrolactone moiety (Mathema et al., 2012). Recent investigations reveal the engagement of several cellular processes including inhibition of NF-κB transcription factor and activation of the p53 pathway. Such promising inhibitory activity has led to a surge of interest in developing structural analogs to improve the cytotoxicity and pharmacological properties of the parent natural product. In a series of reports (Alwaseem et al., 2018; Kolev et al., 2014; Tyagi et al., 2016), the Fasan laboratory has shown the viability of performing a late-stage enzymatic hydroxylation to introduce additional hydroxyl groups at various carbons within parthenolide (Fig. 2B). Similar to their work in artemisinin hydroxylation, they began with P450BM3 variant FL#62, which was found to react with parthenolide to produce a mixture of three products, **6, 7**, and **8** in a 77 : 13 : 10 ratio. Further site-saturation mutagenesis of “first-sphere” active site residues of FL#62 led to the identification of two variants, XII-F12 and VII-H11, which display improved site-selectivity for C9 and C14 hydroxylation, respectively, as well as improved chemoselectivity for hydroxylation over C1, C10-epoxidation. The newly introduced hydroxyl groups were used as a starting point for further scaffold diversification through esterification and carbamoylation. This work resulted in the identification of several analogs with promising antileukemic activity and selectivity for acute myeloid leukemia cells versus healthy cells.

### Divergent Oxidation of Steroids

Steroids are tetracyclic terpenoids that play indispensable roles in many physiological functions. Many steroids, such as progesterone, testosterone, and steroidal bile acids, act as key hormonal compounds and vitamins as well as key regulators in metabolic and immune processes. To date, semisynthetic steroids make up more than 100 Food and Drug Administration-approved therapeutics and several naturally occurring marine steroids (Aiello et al., 1999) have also been found to exhibit potent anti-inflammatory and anti-angiogenic properties. Due to challenges associated with *de novo* chemical synthesis, steroidal drugs have traditionally been prepared via semisynthetic derivatization of naturally abundant steroids. However, chemical methods for remote oxidation of steroids are limited and constitute only a minor fraction of organic reactions that are commonly employed in steroid semisynthesis. With the exception of several template strategies (Breslow, 1995), C–H functionalization of steroids often relies on the use of directed functionalization using a preexisting functional group (Renata et al., 2015).

**Fig. 3.**
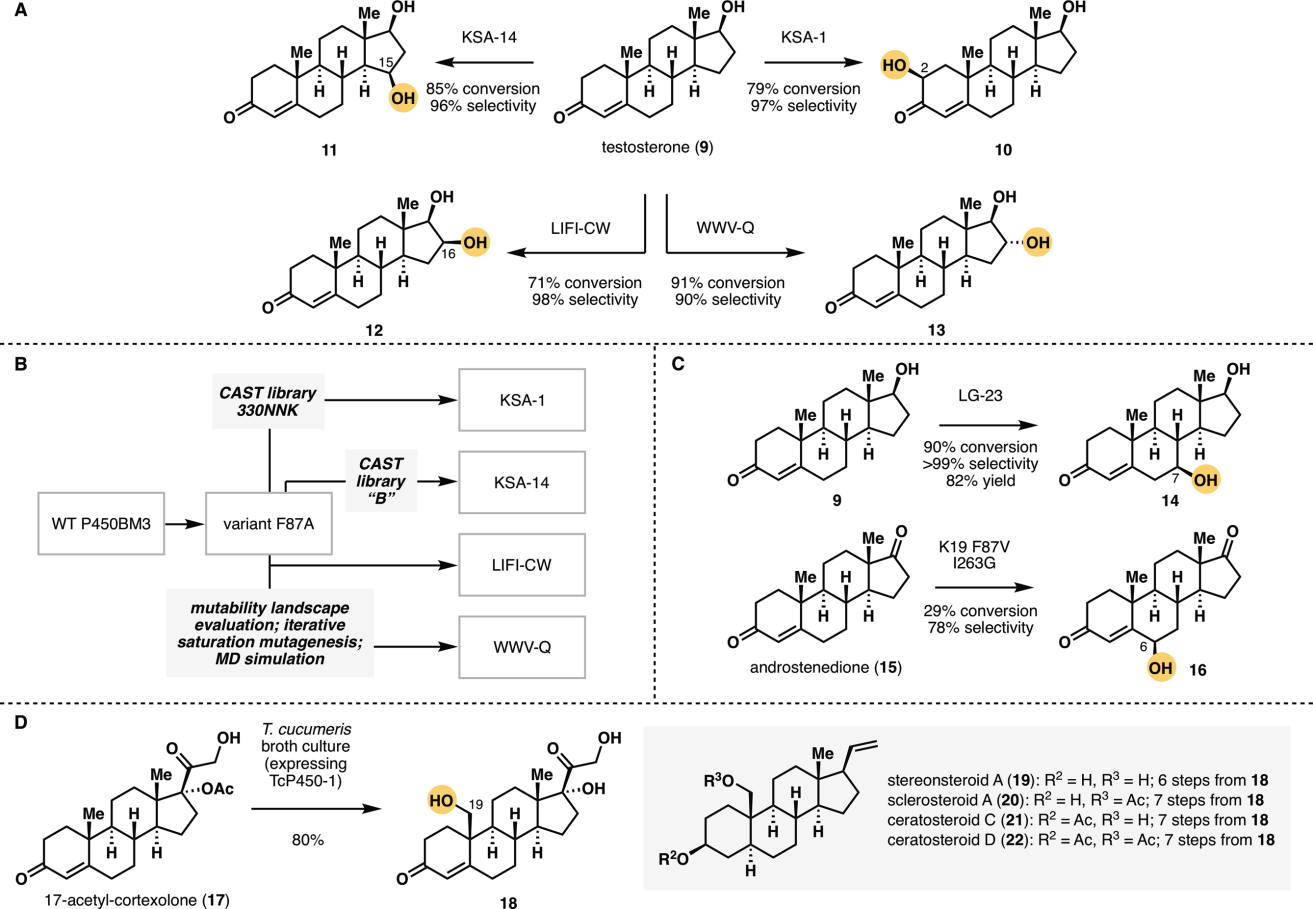
(A) Discovery of P450BM3 variants as biocatalysts for selective C2, C15, and C16 hydroxylation of testosterone (**9**). (B)**.** A brief summary of engineering strategy to generate variants KSA-1, KSA-14, LIFI-CW, and WWV-Q from WT P450BM3; CAST = Combinatorial Active-Site Saturation Test. (C) Recent discovery of P450BM3 variants for selective C-ring hydroxylation of steroids. (D) Recent discovery of biocatalytic C19 hydroxylation of cortexolone acetate (**17**) using P450 from *T. cucumeris*.

Recent investigations into the use of enzymatic hydroxylation have begun to open the possibility of effecting C–H hydroxylation at remote positions on steroidal structures. In 2011, Reetz and coworkers reported (Kille et al., 2011) the engineering of several P450BM3 variants for selective C2 and C15 hydroxylation of testosterone using a structure-guided directed evolution method termed Combinatorial Active-Site Saturation Test (CAST). This method relies on the use of iterative site-saturation mutagenesis on multiple active-site residues to generate focused enzyme libraries, which are then screened for hydroxylation activity on testosterone. Starting with the single mutant P450BM3 F87A, the authors identified several key mutations that are vital for improving reaction conversion and regioselectivity in the hydroxylation of testosterone (**9**, Fig. 3A, B). The best variant for C2 hydroxylation, KSA-1, contains two mutations away from wild-type (WT) enzyme and catalyzes the C2 hydroxylation of testosterone with 97% regioselectivity and 79% conversion. Conversely, a variant called KSA-14 is capable of selective hydroxylation at C15 with 96% regioselectivity and 85% conversion. Using a similar approach but with added incorporation of mutability landscape analysis and molecular dynamics simulation, the Reetz laboratory further identified two variants of P450BM3, LIFI-CW, and WWV-Q for selective production of two diastereomers of C16-hydroxylated product from testosterone (Acevedo-Rocha et al., 2018). Amidst these engineering efforts, concurrent work by several laboratories also led to the discovery of various P450 enzymes that are capable of hydroxylating steroids en route to their catabolism in actinobacteria and protobacteria (Szaleniec et al., 2018). Some of these P450s are able to hydroxylate previously inaccessible sites on steroidal structures and thus represent potentially useful biocatalysts that would complement existing engineered P450BM3 variants for steroid semisynthesis. However, the utility of these P450s in large-scale biotransformation still remains to be reported.

**Fig. 4.**
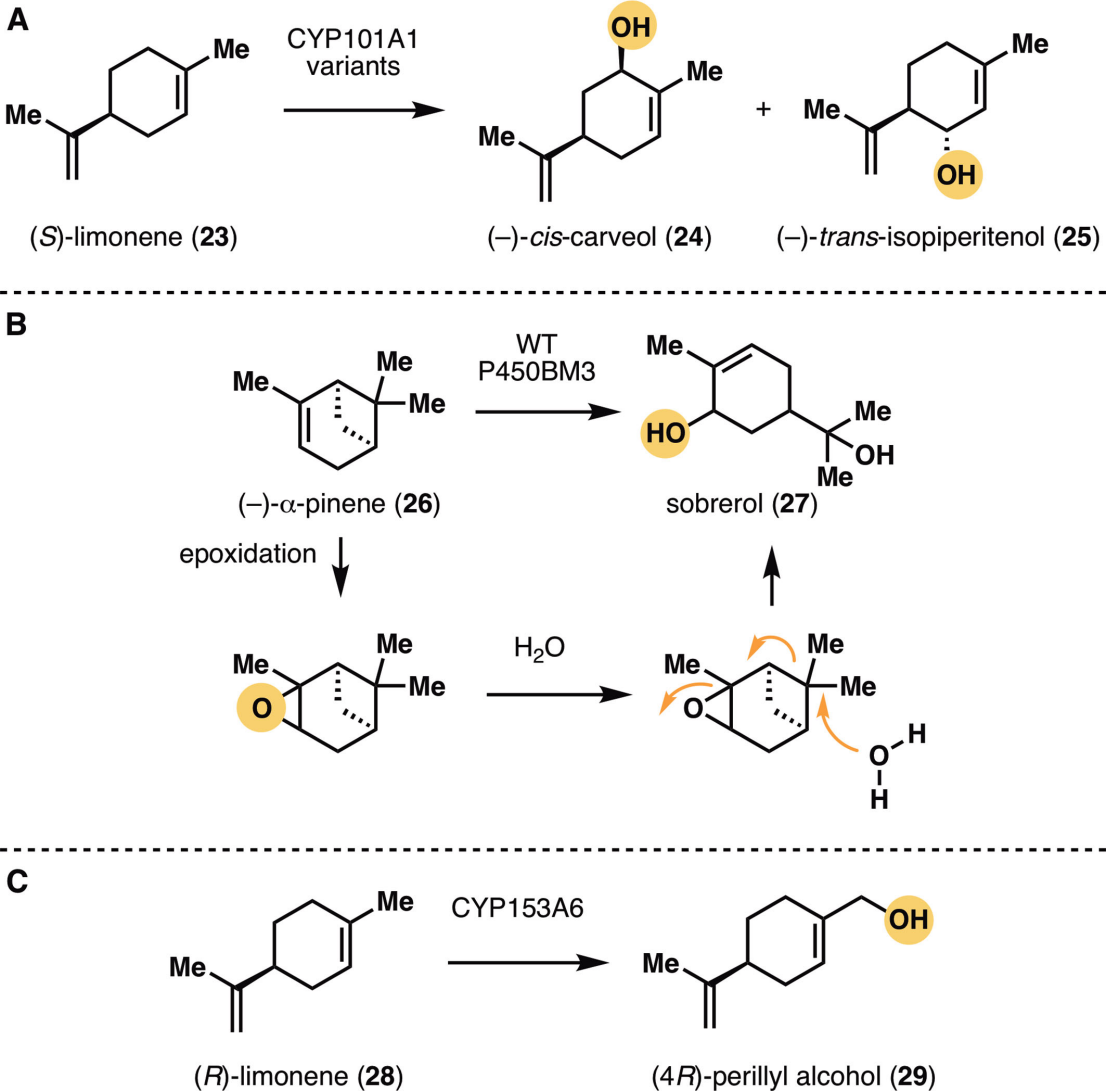
(A) Oxidative modification of (*S*)-limonene (**23**) by CYP101A1 variants. (B) Conversion of (−)-α-pinene (**26**) to sobrerol (**27**) catalyzed by WT P450BM3. (C) CYP153A6-catalyzed conversion of (*R*)-limonene (**28**) to (4*R*)-perillyl alcohol (**29**).

Earlier this year (Li et al., 2020), the Reetz laboratory reported an engineering strategy that led to the discovery of a P450BM3-based steroid C7-hydroxylase (Fig. 3C). C7-hydroxylated steroids are known to possess neuroprotective and anti-inflammatory properties and have also been implicated in immunological processes. However, current chemical routes and biocatalytic processes to access such motif are laborious and low yielding. Starting from a triple mutant (F87G/A328G/A330W) that possesses 3% regioselectivity for C7 hydroxylation of testosterone, 15 residues at or near the active site were identified for mutagenesis. To avoid screening burden associated with multiple site-saturation mutagenesis, single site-saturation mutagenesis library was created for each of these sites and screened individually. The beneficial mutations identified in this initial screening were then rescreened in a combinatorial fashion to identify an optimal combination for C7 hydroxylation of testosterone. This approach yielded a variant called LG-23, which is found to contain 14 mutations away from WT and is capable of hydroxylating various steroids at their C7 carbon with high selectivity and isolated yields. An independent work from Wong and co-workers (Chen et al., 2020) recently showed the utility of glycine scanning mutagenesis in the identification of various P450BM3 variants that display unique regioselectivity profile in steroid hydroxylation.

Efforts to identify suitable P450 catalysts for steroid hydroxylation are not confined only to the prokaryotic P450 space. Recent work (Wang et al., 2019) by Qu and Zhou identified a fungal P450 from *Thanatephorus cucumeris* (TcP450-1) that displays exclusive C19 hydroxylation activity on cortexolone (Fig. 3D). Prior to this work, direct functionalization of the C19 methyl group of steroids is regarded as an unsolved challenge in chemical synthesis and contemporary strategies (Renata et al., 2015) to achieve such functionalization rely on multistep methods. Further optimization of reaction conditions and substrate screening revealed 17-acetyl-cortexolone (**17**) to be a superior substrate with 80% reaction conversion to product **18** following *in situ* hydrolysis of the C17 acetyl group. To demonstrate the synthetic utility and practicality of this biotransformation, the reaction could be conducted on multi-gram scale using *T. cucumeris* culture and the resulting hydroxylated product was taken forward to six C19-hydroxylated steroidal natural products in 4–9 steps.

## Building Block Synthesis

### Monoterpene Hydroxylation

In addition to their use as fragrance components and flavoring agents, monoterpenes have also served as useful building blocks in chemical synthesis (Brill et al., 2017). Commonly referred to as “chiral pool synthesis,” this is a target-oriented synthetic approach that aims to improve synthetic efficiency by looking for full or partial structural match between a cheap, naturally available chiral substance and the intended synthetic target. One of the advantages of using this strategy is the ability to leverage “built-in” chirality in the starting material such that the remaining synthetic sequence can focus on strategic bond construction instead of the identification of suitable asymmetric transformation to set various stereocenters in the final product. This strategy has resulted in many creative and highly efficient total syntheses of terpenoids over the years and still remains a highly active research field (Brill et al., 2017).

Given the widespread use of monoterpenes in chiral pool synthesis, novel methods for their derivatization would provide access to unique building blocks that would subsequently enable more streamlined construction of more complex terpenoids. In 2018, Scrutton and coworkers reported the development of a toolkit for selective oxidation of monoterpenes involving the testing of a library of P450 enzymes in an untargeted GC/MS-based metabolomics screen (Hernandez-Ortega et al., 2018). Prior to this work, efforts on oxyfunctionalization of monoterpenes have largely relied on whole-cell biotransformations and often result in non-selective functionalization. The P450 library used in this work consists of WT CYP101A1 (P450cam) and eight related variants (CYP101A1.1–8), as well as WT P450BM3 and WT CYP153A6. In its native form, CYP101A1 performs selective hydroxylation of camphor (Dus, 1984) while CYP153A6 is known to hydroxylate linear *n*-alkanes (Funhoff et al., 2006). Screening this library for oxidation of fifteen monoterpenes revealed the production of several valuable products. Mutants of CYP101A1 were particularly effective for oxidation of bicyclic monoterpenes and were found to be superior to the WT enzyme, though the reactions commonly produced varying levels of regioselectivity. For example, oxidation of (*S*)-limonene (**23**) with variants CYP101A1.1–3 (Fig. 4A) afforded a mixture of (−)-*cis*-carveol (**24**) and (−)-*trans*-isopiperitenol (**25**). Interestingly, WT P450BM3 was found capable of converting (−)-α-pinene (**26**) to sobrerol (**27**), a known clinical expectorant (Fig. 4B). This transformation was proposed to proceed through initial olefin epoxidation, followed by water-mediated skeletal rearrangement. Several useful reactions were also observed in the reaction of monocyclic monoterpenes with WT CYP153A6 (Fig. 4C), including the production of (4*R*)-perillyl alcohol (**29**) from (*R*)-limonene (**28**).

### C3 Hydroxylation of Sclareolide and Sclareol

Commonly used as fragrance components, sclareolide and sclareol are two inexpensive and abundant cyclic terpenoids that have also seen wide applications as building blocks in terpenoid total syntheses (Dixon et al., 2012; Quinn et al., 2016). Both sclareolide and sclareol contain an embedded trimethyldecalin unit that maps almost exactly onto the structures of many more complex meroterpenoids. This feature allows practitioners of natural product total synthesis to focus their efforts on the construction of the eastern portion of their targets—made possible by the presence of the lactone moiety and terminal allylic alcohol moiety of sclareolide and sclareol, respectively—without having to expend unnecessary chemical steps on construction of the western portion. One notable example of this approach is the development of a unified access to a suite of phenol-containing meroterpenoids through the conversion of sclareolide to “borono-sclareolide” (Dixon et al., 2012). Despite this success, earlier efforts in sclareolide-based semisynthetic approach to meroterpenoids do not permit access to targets containing C3-hydroxylation, which arise in nature via polyene cyclization of linear epoxide precursors. Attempts to effect C–H functionalization on sclareolide using small-molecule-based reagents and catalysts result in predominantly C2 functionalization due to a combination of steric and electronic effects (Chen & White, 2010; Newhouse & Baran, 2011).

**Fig. 5.**
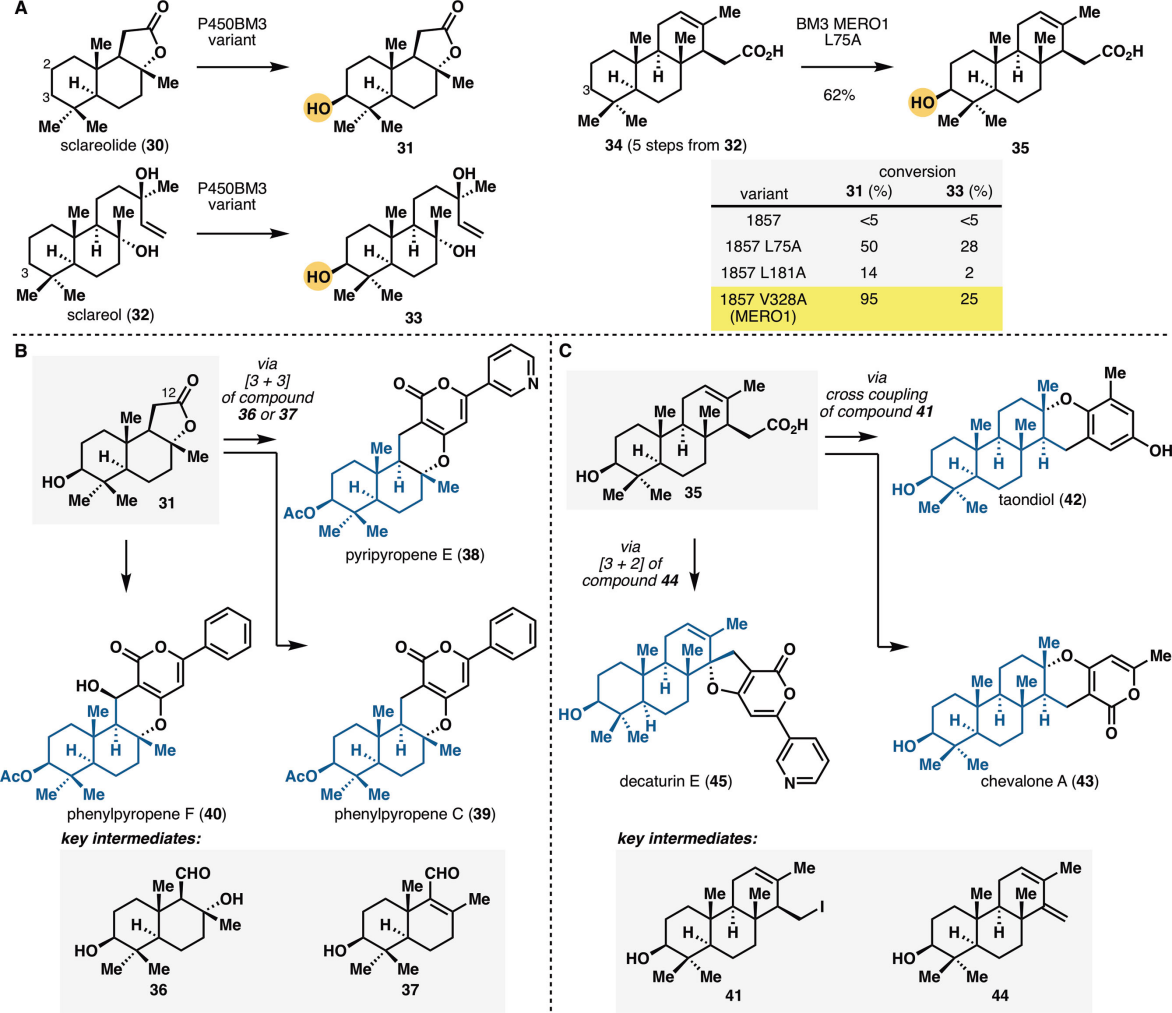
(A) Optimization of biocatalytic C3 hydroxylation of sclareolide (**30**) and sclareol (**32**) and application of MERO1 L75A in the biocatalytic hydroxylation of **34**. (**B**) Divergent synthesis of α-pyrone meroterpenoids from **31**. (**C**) Divergent synthesis of meroditerpenoids from **35**.

In their initial development of fingerprinting-based discovery strategy for terpene hydroxylation biocatalysts (Zhang et al., 2011), Fasan and co-workers disclosed that one of their engineered P450BM3 variants, II-H8, is capable of catalyzing regio- and stereoselective C3 hydroxylation of sclareolide (**30**) in 83% yield (50 mg scale in vitro reaction with 1 mM substrate concentration). Our laboratory saw this as an opportunity to develop a divergent chemoenzymatic access to a wide range of oxidized meroterpenoids that will also address prior limitations in sclareolide-based semisynthetic strategy (Li et al., 2020). We first sought to identify other P450BM3-based catalysts that can affect the same C3 oxidation of sclareolide (**30**) on multi-gram scale at higher substrate concentration (Fig. 5A). A small alanine-scanning library was constructed starting from a P450BM3 variant called 1857, leading to the identification of a key mutation V328A that dramatically increased C2 hydroxylation activity. On gram scale reaction, lysates of *E. coli* expressing 1857 V328A (also called “BM3 MERO1”) was found to afford 60–70% isolated yield of **31** from **30** at 5 mM substrate concentration. Through functional group interconversion of the C12 lactone to generate key intermediate **36** or **37** and coupling with various aromatic fragments (Fig. 5B), we were able to access five different α-pyrone meroterpenoids in 7–11 steps.

To expand the utility of this synthetic strategy further, we turned our focus on the chemoenzymatic synthesis of several oxidized meroditerpenoids from sclareol. While several variants in our P450BM3 library were able to hydroxylate sclareol at its C3 carbon, the reaction conversion proved sub-optimal. As a workaround, we elected to perform substrate engineering to identify a derivative of sclareol that would (i) show superior reaction conversion and (ii) constitute a viable intermediate in the proposed synthesis. To this end, sclareol was converted to acid **34** through a five-step route featuring a radical-based intramolecular cyclization to forge the C8–C14 bond present in our meroditerpenoid targets. Submission of all the intermediates produced in this synthetic route to biocatalytic hydroxylation with our P450BM3 variants revealed acid **34** and variant MERO1 L75A to be the optimal substrate/catalyst combination. This reaction could be carried out on gram-scale with 62% isolated yield of **35** as the sole product. The pendant carboxylic acid of **35** was viewed as a versatile functional handle for the introduction of the aromatic fragments to complete the synthesis of our meroditerpenoid targets (Fig. 5C). Conversion of the carboxylic acid moiety to the corresponding iodide (**41**) was followed by nickel-catalyzed cross-coupling and a final deprotection step to furnish taondiol (**42**) and chevalone A (**43**). The primary iodide moiety could also be eliminated to afford the corresponding terminal methylene (compound **44**), which in turn was submitted to a formal [3 + 2] coupling to complete the synthesis of decaturin E (**45**) and stypodiol. Overall, this chemoenzymatic approach provides rapid synthetic access to eight different meroterpenoids and demonstrates the enabling nature of combining biocatalytic C–H oxidation and radical-based synthetic paradigm in terpenoid construction. In light of the prevalence of hydroxytrimethyldecalin unit in many terpenoids, this report provides the groundwork for future synthetic access to many other terpenoid families using similar strategy.

### Divergent Oxidation of Steviol

**Fig. 6.**
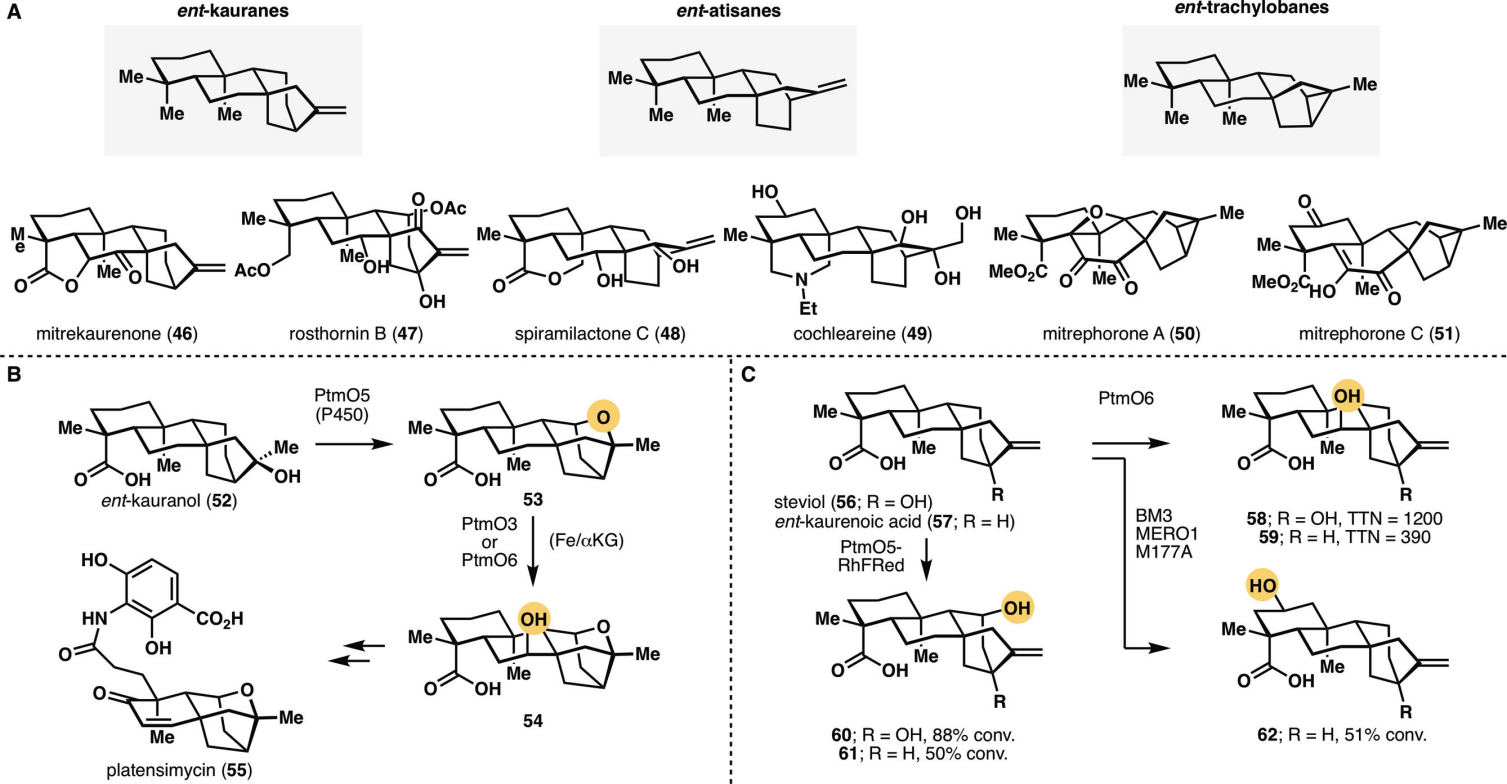
(A) Representative examples of *ent*-kaurane, *ent*-atisane and *ent*-trachylobane natural products. (B) Biosynthesis of platensimycin (**55**) involving oxidative modifications of *ent*-kauranol (**52**) by PtmO5 and PtmO3/O6. (**C**) Biocatalytic A, B, and C ring hydroxylation of steviol (**56**) and *ent*-kaurenoic acid (**57**) with BM3 MERO1 M177A, PtmO6, and PtmO5-RhFRed, respectively.

Derived biosynthetically from *ent*-copalyl pyrophosphate, the *ent*-kauranes, *ent*-atisanes, and *ent*-trachylobanes (Riehl et al., 2015) are three highly diverse families of diterpenoid natural products with intriguing medicinal properties (Fig. 6A). One common characteristic shared by these three families is the multitude of oxygenation patterns present in many of their members. Some of these oxidative processes are also thought to initiate a variety of skeletal rearrangements, contributing to the enormous structural diversity of the families. Due to a lack of understanding of enzymes that are responsible for these oxidative modifications, synthetic biology approaches to produce these three natural product families have witnessed only limited progress. In contrast, many *de novo* chemical synthesis strategies to these natural products have been reported, including several semisynthetic efforts starting from the highly abundant and inexpensive stevioside (Cherney et al., 2014; Kobayashi et al., 2018). However, such semisynthetic approaches suffer from the lack of tools available for remote functionalization of the carbocyclic scaffold. Our laboratory sought to develop a more systematic semisynthetic pursuit of oxidized *ent*-kauranes, *ent*-atisanes and *ent*-trachylobanes by combining contemporary tools of chemical synthesis and biocatalytic strategies for C–H hydroxylation (Zhang et al., 2020). In prior collaborative efforts with the Shen laboratory, we have identified two oxygenases from platensimycin (**55**, Fig. 6B) biosynthesis, PtmO5 and PtmO6, that can effect regio- and stereoselective hydroxylation at C11 and C7 of *ent*-kauranol (**52**), respectively (Rudolf et al., 2018; Dong et al., 2019). Preliminary investigation revealed that both steviol (**56**) and *ent*-kaurenoic acid (**57**) are suitable substrates for hydroxylation with PtmO6 (Fig. 6C). Similarly, PtmO5 (heterologously expressed as a chimeric construct featuring artificial fusion with the reductase domain of P450RhF [Li et al., 2007]) was able to hydroxylate both *ent*-kaurenoic acid and steviol with excellent reaction conversion. In parallel, we also screened our P450BM3 variant library for hydroxylation activity on steviol and *ent*-kaurenoic acid. From these efforts, we observed that variant MERO1 M177A could effect a selective C2 hydroxylation of *ent*-kaurenoic acid exclusively without any observable side reactions. Importantly, all of these hydroxylation reactions could be routinely conducted on gram scale without any observable decrease in isolated yields.

**Fig. 7.**
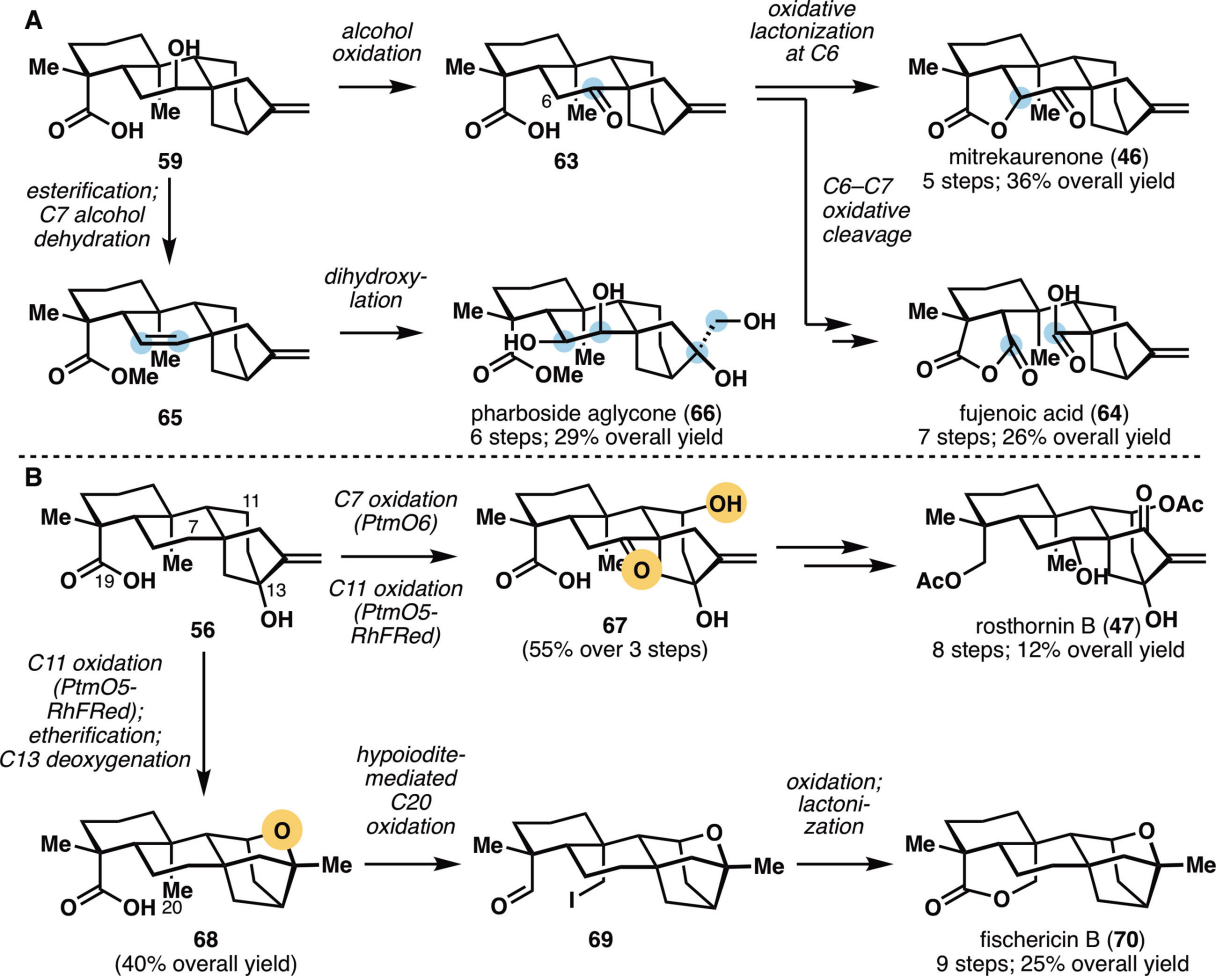
(A) Chemoenzymatic synthesis of mitrekaurenone (**46**), fujenoic acid (**64**), and pharboside aglycone (**66**) starting from **59**. (B) Chemoenzymatic synthesis of rosthornin B (**47**) and fischericin B (**70**) starting from steviol (**56**).

We next showcased the synthetic applications of this biocatalytic hydroxylation platform in the synthesis of several oxidized *ent*-kaurane natural products. As an initial proof of concept, three *ent*-kaurane targets, namely mitrekaurenone, fujenoic acid, and pharboside aglycone, were chosen as they would require only the application of PtmO6 hydroxylation (Fig. 7A). Conversion of *ent*-kaurenoic acid to mitrekaurenone proceeded in three steps consisting of C7 hydroxylation with PtmO6, alcohol oxidation to the ketone and oxidative lactonization at C6. We found that PtmO6 is capable of effecting promiscuous hydroxylation at C6 on several *ent*-kauranes and related substrates when a ketonic moiety is present at C7. Capitalizing on this discovery, we subjected ketone **63** to C6 hydroxylation with PtmO6. Oxidative cleavage of the C6–C7 bond, followed by further oxidation with Dess–Martin periodinane completed the synthesis of fujenoic acid (**64**). Finally, saponification of alcohol **59**, followed by dehydration to diene **65** and global dihydroxylation furnished pharboside aglycone (**66**). This chemoenzymatic strategy could be extended to other targets that would require multiple enzymatic hydroxylations or a combination of enzymatic and chemical hydroxylations (Fig. 7B). For example, by relying on a combination of C7 hydroxylation with PtmO6 and C11 hydroxylation with PtmO5-RhFRed, we successfully prepared rosthornins B (**47**) in eight steps from steviol, respectively. Similarly, by combining C7 hydroxylation with PtmO6 and hypoiodite-based C–H functionalization at C20, we synthesized fischericin B (**70**) in nine steps from steviol.

**Fig. 8.**
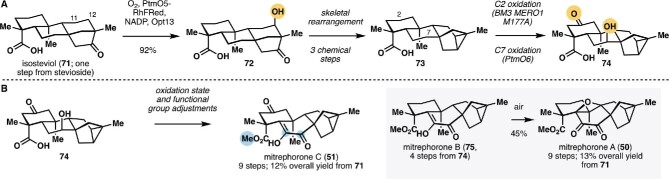
(A) Chemoenzymatic access to *ent*-trachylobane products **73** and **74** starting from isosteviol (**71**). (B)**.** Chemoenzymatic synthesis of the mitrephorones from **74**.

To expand the utility of this platform further, we next targeted the synthesis of the mitrephorones (Li et al., 2005), three *ent*-trachylobane natural products that contain highly unusual oxidation patterns (Fig. 8A). In our design, we envisioned that a C ring hydroxylation of isosteviol (accessible from stevioside in one step) would serve as a gateway to effect a skeletal rearrangement of its C/D ring bicycle to provide an *ent*-atisane product. In turn, a C–C bond formation event between C13 and C16 would produce an *ent*-trachylobane product. The use of PtmO5-RhFRed on isosteviol (**71**) surprisingly led to the formation of **72** instead of the corresponding C11-oxidized product. Though requiring further mechanistic details, one rationale for this observation is that isosteviol's C12 β-H adopts an axial configuration, and therefore has the more appropriate geometry to engage the active Fe(IV)-oxo species relative to the equatorial C11 β-H. This discovery allowed us to design a series of carbocationic rearrangements to furnish *ent*-trachylobane product **73** in three chemical steps. Starting from **73**, consecutive C2 and C7 oxidations with BM3 MERO1 M177A and PtmO6, followed by functional group manipulations and redox adjustments completed the synthesis of mitrephorone C (**51**, Fig. 8B). By omitting the C2 oxidation step, the same approach could be used to provide mitrephorone B (**75**). During this campaign, we also discovered that mitrephorone B is capable of undergoing slow autooxidation to produce mitrephorone A (**50**).

**Fig. 9.**
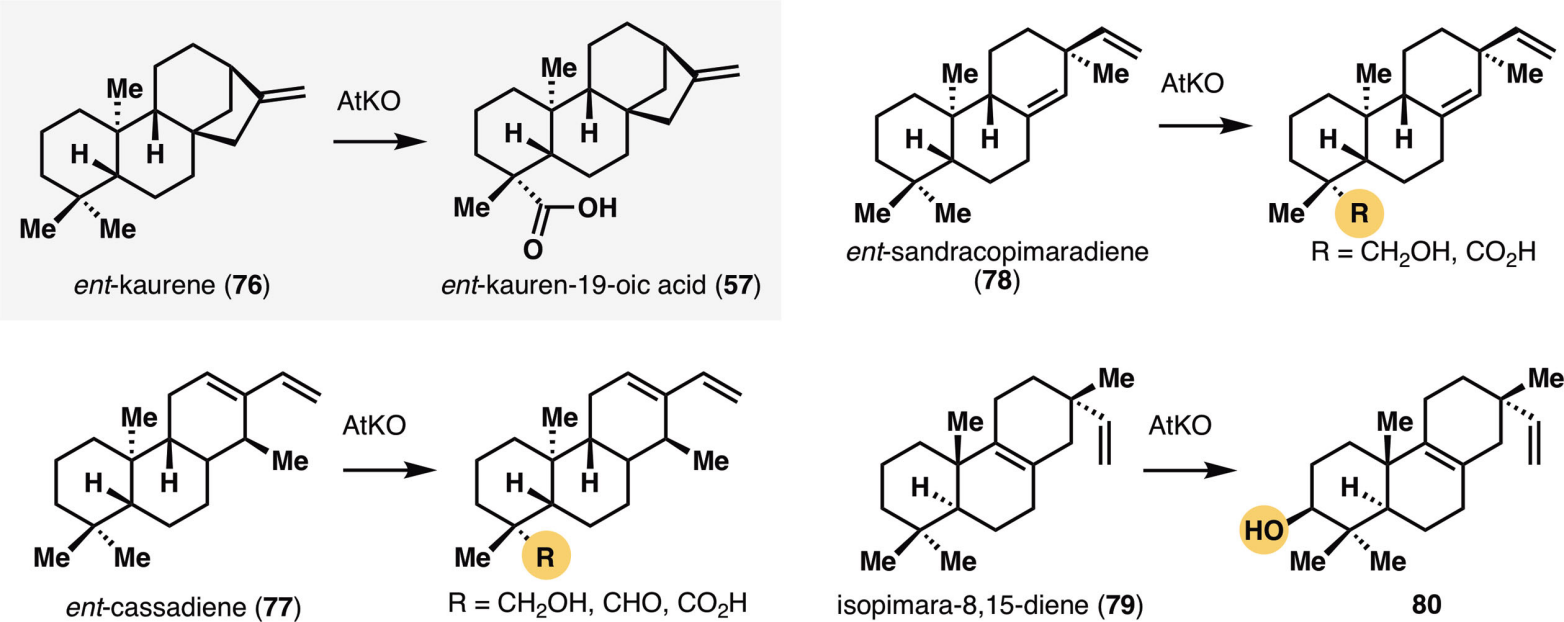
Hydroxylation of *ent*-cassadiene (**77**), *ent*-sandracopimaradiene (**78**), and isopimara-8,15-diene (**79**) by AtKO (*A. thaliana* kaurane oxidase).

## Conclusion and Future Directions

Biocatalytic oxidation holds tremendous potential for more efficient access to bioactive and medicinally relevant terpenoids. In particular, the ability to introduce key hydroxyl groups in a site-selective fashion addresses many unmet challenges in contemporary natural product total synthesis approaches. In light of these features, biocatalytic oxidation methods have attracted a lot of interests in recent years. The research highlighted in this review illustrates recent progress that has been made in the development of practical biocatalytic C–H hydroxylation approaches for terpenoid functionalization through engineering of existing oxygenases or exploration of new enzymes. In all of these cases, access to novel terpenoid derivatives or more efficient synthesis of complex terpenoids could be obtained, demonstrating the power and utility of this approach, especially when combined with contemporary tools in organic synthesis. Though encouraging, there are still key challenges that need to be addressed in order to propel the field further. First, not all biocatalytic oxidation methods exhibit synthetically useful selectivity profile and reaction conversion/yield. This issue commonly arises either due to the poor instability of the enzyme used in the reaction or the inherently poor kinetic parameters of the enzyme. While such factors could be remedied through the use of protein engineering and directed evolution strategies (Galanie et al., 2020), these endeavors still rely very much on the use of relatively low throughput analytical methods such as gas chromatography (GC) or liquid chromatography/mass spectrometry (LC/MS) analyses. In this regard, the field would benefit greatly from the development of novel high-throughput screening strategies (Holland-Moritz et al., 2020).

In addition, there is still relatively limited number of enzymes available in our arsenal for terpenoid oxidation. With advances in sequencing technology, new oxygenases are discovered almost on a regular basis. However, such discovery is typically performed in the context of biosynthetic gene cluster elucidation and it is uncommon to see concurrent exploration of the biocatalytic utility of the enzyme in context. It is becoming widely accepted that most, if not all, enzymes exhibit some levels of catalytic and substrate promiscuity and at times, investigation into this property may lead to unexpected but useful discovery. This idea is highlighted in a recent study on the substrate specificity of *ent*-kaurane oxidases in the modification of more than 20 terpenoid substrates (Mafu et al., 2016). While conventional wisdom may predict that these oxidases would be highly specific for *ent*-kaurane substrates, Peters and coworkers observed that a kaurene oxidase from *A. thaliana* exhibits surprising cross-reactivity with a range of terpenoid substrates such as *ent*-cassadiene and isopimara-8,15-diene (Fig. 9). Still few and far between, similar systematic investigations on the substrate promiscuity of other oxygenases with a wide range of terpenoid substrates could result in more useful discoveries for biotechnological and synthetic applications.

Finally, there has been a lot of emphasis to date in the use of the P450s for biocatalytic oxidation. However, P450s are known to suffer from several key limitations due to their inherent structural and catalytic features, including the need for appropriate reductase partner proteins and the presence of transmembrane domains in many eukaryotic P450s (Fasan, 2012). Recent studies have shown that other oxygenase superfamilies (Baker Dockrey & Narayan, 2019; Zwick & Renata, 2020) are just as adept as the P450s for scalable and practical oxidative modifications of many small molecule scaffolds while also possessing certain advantages, such as the lack of requirements for reductase partners. Given the relative lack of biocatalytic and biotechnological applications of these oxygenases, we believe that their exploration in terpene modifications will be a fertile area to mine for new discoveries.
